# Preconception Care: A Strategic Intervention for the Prevention of Neonatal and Birth Disorders

**DOI:** 10.7759/cureus.41141

**Published:** 2023-06-29

**Authors:** Harshal Khekade, Ashwini Potdukhe, Avinash B Taksande, Mayur B Wanjari, Seema Yelne

**Affiliations:** 1 Community Medicine, Jawaharlal Nehru Medical College, Datta Meghe Institute of Higher Education & Research, Wardha, IND; 2 Medical Surgical Nursing, Smt. Radhikabai Meghe Memorial College of Nursing, Datta Meghe Institute of Higher Education & Research, Wardha, IND; 3 Physiology, Jawaharlal Nehru Medical College, Datta Meghe Institute of Higher Education & Research, Wardha, IND; 4 Research and Development, Jawaharlal Nehru Medical College, Datta Meghe Institute of Higher Education & Research, Wardha, IND; 5 Nursing, Shalinitai Meghe College of Nursing, Datta Meghe Institute of Higher Education & Research, Wardha, IND

**Keywords:** maternal and fetal health, preventive healthcare, intervention, birth disorders, neonatal health, preconception care

## Abstract

Preconception care is a strategic intervention to improve neonatal and birth outcomes by addressing modifiable risk factors and optimizing maternal and fetal health before pregnancy. This review article examines the importance of preconception care and its impact on preventing neonatal and birth disorders. The methodology involved a comprehensive review of peer-reviewed articles, research studies, and authoritative reports. Key components of preconception care, evidence-based interventions, and their effectiveness in reducing specific neonatal and birth disorders are discussed. The review also highlights the challenges and barriers in implementing preconception care, such as lack of awareness, socioeconomic factors, health system limitations, and policy considerations. Strategies for promoting preconception care, including integration into healthcare systems, raising awareness, healthcare professional education, and collaborations are presented. The conclusion emphasizes the significance of preconception care as a strategic intervention and calls for action by healthcare providers, policymakers, and individuals to prioritize preconception care for better neonatal and birth outcomes.

## Introduction and background

Preconception care refers to healthcare interventions and services provided to individuals or couples before pregnancy to optimize their health and promote favorable outcomes for both the mother and the baby. It recognizes that the period preceding conception is critical for the health and development of the future child. Preconception care encompasses a range of interventions, including health promotion, risk assessment, and management of pre-existing conditions [1].

In recent years, there has been growing recognition of the significance of preconception care in preventing neonatal and birth disorders. Research has shown that individuals' health and lifestyle choices before conception can significantly influence the health outcomes of their offspring [2]. By addressing modifiable risk factors and providing targeted interventions, preconception care can reduce the incidence of various neonatal and birth disorders, leading to healthier pregnancies and improved long-term health outcomes for children [2]. Neonatal and birth disorders encompass many conditions that can have lifelong consequences for individuals and their families. These disorders can manifest as physical, cognitive, or developmental abnormalities and may significantly impact affected individuals' well-being and quality of life [3].

The global burden of neonatal and birth disorders is substantial, with 2.4 million babies affected yearly [4]. These conditions can increase mortality rates, long-term disabilities, and healthcare costs. Furthermore, they place emotional, financial, and social burdens on families and communities [4]. Addressing neonatal and birth disorders requires a multifaceted approach that spans the entire continuum of care, including preconception, prenatal, and postnatal periods. Preconception care is vital in this continuum by targeting modifiable risk factors and promoting optimal health before pregnancy [5]. This review article examines the role of preconception care as a strategic intervention for preventing neonatal and birth disorders. By exploring the existing literature and evidence, we aim to shed light on the impact of preconception care on improving maternal and fetal health outcomes.

## Review

Methodology

The review employed a comprehensive research approach, including peer-reviewed articles, research studies, and authoritative reports. The databases used for the search included PubMed, Embase, and Scopus. The selection criteria were designed to ensure the inclusion of high-quality, appropriate, and evidence-based literature. Both electronic databases and manual searching techniques were used to gather relevant information on preconception care and its impact on neonatal and birth outcomes. The selection criteria ensured the inclusion of high-quality, appropriate, and evidence-based literature. Studies addressing preconception care, its components, interventions, and its impact on neonatal and birth outcomes were included. Various study designs were considered and published in English within a specific timeframe. Only peer-reviewed articles were included, while case reports and editorials were excluded. While case reports provide valuable insights into individual cases, they are generally limited in generalizability. They may not provide a robust evidence base for drawing broader conclusions. The decision to exclude editorials strictly focused on primary research studies and peer-reviewed articles, which are typically subjected to rigorous evaluation and quality assessment.

Preconception care: concept and definition

Definition and Scope of Preconception Care

Preconception care refers to interventions and services provided to individuals or couples before conception to optimize their health and well-being, aiming to improve pregnancy outcomes and the future child's health. It involves a proactive and comprehensive approach that addresses various aspects of health, including physical, mental, and social well-being. The scope of preconception care is broad and encompasses a range of interventions and services to improve individuals' or couples' health and well-being before they conceive a child. It goes beyond traditional reproductive health services and takes a proactive approach to address various aspects of health [2-4].

One important aspect of preconception care is health promotion and education. Individuals receive information and guidance about healthy lifestyle choices, such as maintaining a balanced diet, exercising regularly, managing stress, and avoiding harmful substances like tobacco, alcohol, and drugs. This empowers individuals to make informed decisions and adopt behaviors that support their overall health and increase their chances of a healthy pregnancy. Another crucial component of preconception care is the screening and management of medical conditions. Individuals are screened for existing medical conditions, such as diabetes, hypertension, or infectious diseases, and appropriate management strategies are implemented to optimize their health before pregnancy. Addressing these conditions early on can minimize potential risks to the individual and the future child [2,5].

Genetic counseling and testing are also part of preconception care. Couples may receive counseling and, if necessary, undergo genetic testing to assess the risk of inherited genetic disorders or explore family planning options. This allows them to make informed decisions about their reproductive choices and take appropriate steps to mitigate potential risks. Immunizations are an important aspect of preconception care as well. Individuals are evaluated for the need for vaccinations to protect against infectious diseases, ensuring their health and reducing the risk of transmission to the fetus during pregnancy. Ensuring individuals are current with their immunizations can significantly reduce the risk of preventable infections during pregnancy [6].

Rationale for Focusing on the Preconception Period

The preconception period, which refers to the time before conception occurs, is a crucial window of opportunity for improving maternal and fetal health outcomes. It recognizes that the health status of both parents at the time of conception can significantly influence the development and well-being of the fetus. By addressing potential risk factors and optimizing health before pregnancy, preconception care aims to create a favorable environment for conception, implantation, and embryonic development. Various physiological processes are vital for the embryo's development during the preconception period. These include important stages of organ formation and fetal growth, which occur early in pregnancy, often before a woman even realizes she is pregnant. Some of these critical developmental periods have passed by the time prenatal care begins. Therefore, it becomes crucial to intervene during the preconception period to mitigate risks and promote healthy development from the earliest stages [7].

By focusing on the preconception period, healthcare providers and individuals can address potential risk factors that could negatively impact pregnancy outcomes and the future child's health. This may involve interventions such as optimizing nutrition, managing chronic medical conditions, ensuring vaccinations are current, identifying and addressing genetic risks, and promoting healthy lifestyle choices. Addressing these factors and optimizing health before pregnancy can significantly contribute to the well-being of both the mother and the developing fetus. It can reduce the risk of pregnancy complications, birth defects, and other adverse outcomes. Moreover, by taking steps to improve maternal health before conception, there is the potential for long-lasting effects on the health and well-being of the child throughout their life [8].

Key Components of Preconception Care

Health promotion and risk assessment: Preconception care emphasizes promoting healthy behaviors and mitigating health risks. This includes encouraging individuals to maintain a balanced diet, engage in regular physical activity, and avoid harmful substances like smoking, alcohol, and illicit drugs. Additionally, preconception care involves assessing individuals' health risks and providing appropriate guidance and interventions to address and mitigate these risks [9].

Family planning and contraception: Preconception care supports individuals and couples in making informed decisions about family planning. It involves providing access to effective contraception methods and discussing contraceptive options. By addressing any concerns or barriers to family planning, preconception care helps individuals and couples plan for when to start or expand their families in a way that aligns with their reproductive goals [10].

Screening and managing pre-existing conditions: Preconception care focuses on identifying and managing pre-existing medical conditions that may impact pregnancy outcomes. This includes screening for conditions such as diabetes, hypertension, or infections. By identifying these conditions early on, appropriate management strategies, such as medication adjustments, lifestyle modifications, or referrals to specialists, can be implemented to optimize health before pregnancy [11].

Genetic counseling and screening: Preconception care recognizes the importance of assessing the risk of inherited genetic conditions. Genetic counseling is provided to individuals or couples to discuss their genetic testing options and make informed decisions about family planning. This aspect of preconception care ensures that individuals have the necessary information and support to understand and address potential genetic risks [12].

Immunizations and infectious disease prevention: Preconception care highlights the significance of immunizations in protecting individuals and their future children from preventable infections. It ensures that individuals are up to date on essential vaccinations, such as rubella and hepatitis B, which can adversely affect pregnancy. Preconception care also guides preventing and managing infections that may pose risks during pregnancy, helping to create a healthy environment for conception and fetal development [13].

Psychosocial support and mental health considerations: Preconception care acknowledges the importance of psychosocial well-being and mental health. It involves addressing emotional well-being, supporting stress management, and screening for mental health conditions. Preconception care also includes appropriate referrals for counseling or treatment if needed, aiming to help individuals achieve optimal mental health before conception [14].

Importance of Preconception Care for Maternal and Fetal Health

Improved pregnancy outcomes: Engaging in preconception care has been associated with a reduced risk of complications such as preterm birth, low birth weight, and congenital disabilities [6]. By addressing potential risk factors before conception, individuals can optimize their health and increase the chances of a healthy pregnancy and favorable birth outcomes [6].

Enhanced maternal health: Preconception care allows for identifying and managing pre-existing conditions, reducing the risks associated with chronic diseases during pregnancy. By addressing conditions such as diabetes, hypertension, or thyroid disorders before conception, individuals can optimize their health and reduce the likelihood of complications during pregnancy [15].

Identification and management of modifiable risk factors: Through preconception care, lifestyle factors that can impact pregnancy outcomes, such as smoking, excessive alcohol consumption, or inadequate nutrition, can be assessed and addressed. By providing guidance and support, preconception care enables individuals to make positive changes to their lifestyle, reducing the risks associated with these modifiable factors [16].

Early detection and intervention: Preconception care involves screening and counseling to identify potential risks and enable timely interventions. By assessing factors such as genetic disorders, infectious diseases, or mental health conditions, preconception care can facilitate early detection and management of these conditions before pregnancy. This proactive approach allows for appropriate interventions and reduces the potential impact on pregnancy outcomes [17].

Preconception care and the prevention of neonatal and birth disorders

Link Between Preconception Care and Improved Neonatal and Birth Outcomes

Extensive research has demonstrated a strong association between preconception care and improved neonatal and birth outcomes. By addressing modifiable risk factors and promoting optimal health before conception, preconception care can significantly reduce the incidence of various neonatal and birth disorders. The link between preconception care and improved outcomes highlights the importance of implementing effective interventions and strategies in this critical period [18].

Evidence-Based Interventions and Strategies in Preconception Care

Nutritional interventions and supplementation: Nutritional interventions and supplementation play a crucial role in preconception care. Adequate nutrition before conception, including folic acid supplementation, has reduced the risk of neural tube and other congenital disabilities. To support optimal maternal and fetal health, ensuring a balanced diet rich in essential nutrients, such as iron and calcium, is important [19]. 

Lifestyle modifications and behavior changes: Promoting lifestyle modifications and behavior changes is an integral part of preconception care. Encouraging individuals to quit smoking, reduce alcohol consumption, and avoid illicit drug use can significantly decrease the risk of adverse pregnancy outcomes. Similarly, promoting regular physical activity and maintaining a healthy weight is essential for overall health and reducing the risk of complications during pregnancy [20].

Screening and management of pre-existing medical conditions: Screening and managing pre-existing medical conditions are critical components of preconception care. Identifying and managing conditions like diabetes, hypertension, and thyroid disorders before conception can minimize risks to the mother and the fetus. Optimizing medication regimens, providing appropriate counseling, and offering support for managing chronic conditions are essential aspects of preconception care [21].

Genetic counseling and screening: Genetic counseling and screening are important considerations in preconception care. Genetic counseling helps individuals understand their risk of having a child with a genetic disorder and enables them to make informed decisions about family planning. Genetic screening allows for identifying carrier status for specific genetic conditions, empowering couples to make informed reproductive choices [22].

Immunizations and infectious disease prevention: Immunizations and contagious disease prevention are vital in preconception care. Ensuring individuals are current on recommended vaccinations, such as rubella and hepatitis B, protects against infections that can pose risks during pregnancy. Guiding in preventing and managing infections, including sexually transmitted infections, is crucial for protecting maternal and fetal health [23].

Psychosocial support and mental health considerations: Psychosocial support and addressing mental health are essential to preconception care. Addressing psychosocial factors and mental health conditions before conception promotes emotional well-being and reduces the risk of adverse pregnancy outcomes. Providing support and appropriate referrals for counseling or treatment can help individuals manage stress, anxiety, or depression, ultimately contributing to better overall reproductive health [24].

Impact of Preconception Care on Specific Neonatal and Birth Disorders

Neural tube defects: Neural tube defects, such as spina bifida and anencephaly, can be significantly reduced through preconception care interventions. Promoting folic acid supplementation before conception and encouraging a diet rich in folate can effectively decrease the risk of neural tube defects in newborns. Adequate folate intake before pregnancy has been shown to positively impact the development of the neural tube, reducing the occurrence of these birth defects [25].

Congenital heart defects: Preconception care interventions are crucial in reducing the risk of congenital heart defects. Managing pre-existing conditions like diabetes, optimizing maternal weight, and avoiding medications that may have teratogenic effects are important strategies to help lower the incidence of congenital heart defects in newborns. Addressing these factors before pregnancy can mitigate the risk of congenital heart defects [26]. 

Preterm birth: Preconception care addresses various risk factors associated with preterm birth. By targeting behaviors such as smoking and substance abuse and managing chronic medical conditions like hypertension or diabetes, preconception care can help reduce the likelihood of preterm birth. Taking steps to optimize maternal health and addressing these risk factors before conception can improve neonatal outcomes and reduce the preterm birth rate [27].

Low birth weight: Optimizing maternal health is essential for preventing low birth weight babies. Preconception care interventions that focus on providing optimal nutrition, promoting smoking cessation, and managing maternal medical conditions can significantly reduce the risk of delivering babies with low birth weight. By addressing these factors before pregnancy, preconception care aims to create a healthier environment for fetal development, leading to improved birth weight outcomes [28].

Genetic disorders: Preconception care offers genetic counseling and screening opportunities, allowing individuals to assess their hereditary risks and make informed decisions about family planning. By identifying genetic disorders in advance, couples can take preventive measures to minimize the risk of passing on certain genetic conditions to their children. This may involve exploring options such as assisted reproductive technologies or prenatal genetic testing to make informed choices about family planning and reduce the likelihood of having a child with a genetic disorder [29].

Other relevant conditions: Preconception care interventions have shown promise in reducing the risk of other pregnancy-related diseases. For example, addressing risk factors through preconception care can help decrease the incidence of conditions like preeclampsia, gestational diabetes, and intrauterine growth restriction, promoting healthier pregnancies and better birth outcomes [30]. The impact of preconception care on these specific neonatal and birth disorders highlights its significant role in preventing adverse outcomes and promoting healthier pregnancies. Implementing evidence-based interventions and strategies tailored to individual needs can substantially improve maternal and fetal health.

Challenges and barriers to implementing preconception care

Lack of Awareness and Knowledge Among Healthcare Providers and Individuals

The lack of awareness and knowledge among healthcare providers and individuals presents a critical challenge when implementing effective preconception care. Many healthcare professionals may not prioritize preconception care as an essential component of reproductive healthcare and may not have received adequate training. As a result, they may lack a comprehensive understanding of the significance of preconception care or the necessary skills to deliver these services. This knowledge gap among healthcare providers can hinder the integration and delivery of preconception care, leading to missed opportunities for providing crucial patient guidance and interventions. Furthermore, the limited training in preconception care during healthcare providers' education and professional development exacerbates the issue. Without specific training on preconception care, healthcare professionals may not feel confident or equipped to address preconception health effectively. This can contribute to a lack of emphasis on preconception care in clinical practice, preventing individuals from receiving the necessary support and interventions before pregnancy [27-31].

Individually, there is often a lack of awareness about the importance of preconception care. Many individuals may not be aware that taking proactive steps before pregnancy can significantly impact their health and the health of their future children. Moreover, individuals may not have access to accurate information about preconception care, including its benefits and available services. This lack of awareness and access further impedes individuals from seeking preconception care and capitalizing on its potential advantages [30-31]. To address this challenge, efforts should be made at various levels. Firstly, it is crucial to raise awareness among healthcare providers about the significance of preconception care and its integration into routine clinical practice. This can be achieved through targeted professional education, training programs, and developing guidelines emphasizing preconception care's importance. By equipping healthcare providers with the necessary knowledge and skills, they can confidently incorporate preconception care into their practice and provide appropriate guidance to patients [31].

Simultaneously, public awareness campaigns and educational initiatives are essential to increase individuals' understanding of preconception care. This can involve disseminating accurate information through various channels, such as websites, brochures, and public health campaigns. Additionally, community outreach programs can be implemented to provide access to preconception care services and engage with individuals directly. By empowering individuals with knowledge about preconception care, they can make informed decisions and actively seek these services to optimize their health and the health of their future children [31].

Socioeconomic and Cultural Factors Influencing Access to Preconception Care

Socioeconomic and cultural factors significantly impact access to preconception care services. Disparities in healthcare access, affordability, and insurance coverage can create barriers that limit an individual's ability to seek preconception care. Socioeconomic factors such as poverty, low educational attainment, and a lack of social support hinder preconception care access and utilization. Additionally, cultural beliefs, norms, and practices can influence individuals' perceptions of preconception care and willingness to engage. These factors contribute to disparities in access to preconception care, as individuals from disadvantaged backgrounds may face challenges in accessing healthcare facilities, affording the cost of services, or prioritizing preconception care due to competing priorities. Lack of social support and cultural misconceptions about the importance of preconception care can also deter individuals from seeking these services. Addressing these barriers requires targeted efforts to reduce healthcare disparities, improve affordability and insurance coverage, provide education and awareness about the benefits of preconception care, and promote culturally sensitive approaches to ensure equitable access for all individuals and communities [32].

Health System Challenges and Limitations

Fragmented healthcare systems: Fragmentation refers to the lack of coordination and integration between healthcare providers and settings involved in delivering preconception care. This fragmentation can hinder the provision of comprehensive care as individuals may receive services from multiple healthcare providers or locations without proper communication and coordination. The lack of seamless information sharing and care coordination can lead to gaps in care, duplication of services, and missed opportunities for timely interventions [33].

Limited resources: Insufficient funding, infrastructure, and staffing pose significant challenges to the availability and accessibility of preconception care services. Inadequate financial resources can restrict the development and implementation of comprehensive preconception care programs. Limited infrastructure, such as clinics and healthcare facilities, can result in limited access to preconception care services, especially in underserved areas. Additionally, a shortage of healthcare professionals trained in preconception care can limit the capacity to provide comprehensive and timely care to individuals seeking preconception services [34].

Time constraints: Healthcare providers often face time constraints during patient visits, making it challenging to address preconception care needs adequately. The limited time available for patient consultations may result in incomplete discussions about preconception care, leading to missed opportunities for risk assessment, health promotion, and counseling. Adequate time is necessary to provide thorough education, assess individual risk factors, discuss reproductive plans, and develop personalized preconception care plans [35].

Inadequate data collection and monitoring: The availability of comprehensive data collection and monitoring systems related to preconception care is crucial for assessing the impact and effectiveness of interventions. However, the current data collection and monitoring systems for preconception care may be inadequate or non-existent in some healthcare settings. This limitation makes it difficult to track and evaluate preconception care interventions' outcomes, identify improvement areas, and inform evidence-based practices. Without robust data collection and monitoring systems, it is challenging to measure the success of preconception care programs and allocate resources effectively [36].

Addressing challenges: Addressing these challenges requires addressing fragmentation through improved care coordination, increasing funding and resources allocated to preconception care, addressing time constraints by prioritizing preconception care within healthcare visits, and developing comprehensive data collection and monitoring systems. By overcoming these challenges, healthcare systems can enhance the delivery of preconception care and improve neonatal and birth outcomes [37].

Policy and Advocacy Considerations

Lack of specific guidelines and recommendations refers to the absence of clear and standardized guidelines and advice on preconception care at the national or international level. Without established guidelines, healthcare providers may lack clear direction in providing comprehensive preconception care, leading to inconsistent practices and inadequate attention to this critical area of healthcare. The absence of guidelines can result in variations in the quality and scope of preconception care services provided to individuals and couples [38].

Limited policy support and prioritization highlight the challenges faced in garnering sufficient attention and support for preconception care from policymakers. In some healthcare systems, preconception care may not be given the necessary priority or resources, resulting in insufficient funding, infrastructure, and healthcare personnel dedicated to delivering preconception care services. This lack of policy support and prioritization can hinder the establishment and sustainability of preconception care programs and initiatives [39].

Advocacy and public awareness play a crucial role in promoting the importance of preconception care. Advocacy efforts involve raising awareness among policymakers, healthcare providers, and the general public about the significance of preconception care for improving maternal and fetal health outcomes. Increased public awareness can help generate support and drive policy changes prioritizing preconception care. Through advocacy, resources can be allocated, infrastructure can be strengthened, and healthcare systems can be encouraged to integrate preconception care into routine healthcare services. By fostering public awareness, individuals and couples can be empowered to prioritize preconception care and seek appropriate services and interventions to optimize their reproductive health [40].

Addressing these challenges and barriers requires a multi-faceted approach involving the education and training of healthcare providers, targeted outreach and education for individuals, addressing socioeconomic and cultural barriers, strengthening health systems, and advocating for policies prioritizing preconception care as a critical component of comprehensive reproductive healthcare.

Strategies for promoting preconception care

Integration of Preconception Cares Into Existing Healthcare Systems

To promote preconception care effectively, it is essential to integrate it into existing healthcare systems. This involves incorporating preconception care guidelines, protocols, and assessments into routine clinical practice. By embedding preconception care within primary care, family planning, and reproductive health services, healthcare providers can systematically address preconception health needs during patient interactions. Integration facilitates identifying at-risk individuals, providing appropriate interventions, and seamless continuity of care from preconception to pregnancy [41].

Targeted Interventions and Campaigns to Raise Awareness

Raising awareness about the importance of preconception care is vital to ensure its uptake. Targeted interventions and campaigns can be developed to educate individuals, couples, and communities about the benefits of preconception care and the available resources. These interventions can include educational materials, social media campaigns, community outreach programs, and partnerships with community organizations. By disseminating accurate information and emphasizing the impact of preconception care on maternal and fetal health outcomes, awareness can be increased, encouraging individuals to seek preconception care services [42].

Education and Training for Healthcare Professionals

Enhancing the knowledge and skills of healthcare professionals is crucial for successfully implementing preconception care. Educational initiatives should focus on increasing awareness about preconception care, providing evidence-based guidelines and best practices, and training healthcare providers on how to incorporate preconception care into their practice. Continuing education programs, workshops, conferences, and online resources can be utilized to disseminate information and train healthcare professionals on the latest developments in preconception care. By equipping healthcare providers with the necessary knowledge and skills, they can effectively deliver preconception care services and support their patients [43].

Collaborations and Partnerships for Comprehensive Preconception Care

Comprehensive preconception care requires collaboration and partnerships across various sectors. Healthcare providers, public health agencies, community organizations, and policymakers must work together to create a supportive environment for preconception care. Collaborations can involve sharing resources, expertise, and best practices and establishing referral networks and care coordination mechanisms. Partnerships with community organizations can help reach underserved populations and address social determinants of health that impact preconception care access. Engaging policymakers is essential to advocate for policies that support and prioritize preconception care within healthcare systems and allocate resources accordingly [44]. By implementing these strategies, preconception care can be promoted effectively, leading to increased uptake and improved health outcomes for individuals and their future children. Integrating existing healthcare systems, raising awareness, educating healthcare professionals, and fostering collaborations are key steps toward ensuring preconception care becomes an integral part of routine healthcare and reproductive health services.

## Conclusions

In conclusion, the importance of preconception care cannot be overstated, and it requires the collective efforts of healthcare providers, policymakers, and individuals to prioritize and implement it effectively. Healthcare providers play a crucial role in promoting preconception care. It is imperative for them to integrate preconception care into their routine clinical practice. This involves incorporating discussions about preconception health during regular check-ups and providing appropriate guidance and support to individuals of reproductive age. Additionally, healthcare providers should receive comprehensive training and education on the significance of preconception care, its potential benefits, and the necessary steps to ensure its successful implementation. By doing so, they can effectively address the unique needs and concerns of their patients in the preconception period. Equally important is the involvement of policymakers in recognizing preconception care as a critical component of comprehensive reproductive healthcare. Policymakers should acknowledge the importance of preconception care and advocate for its integration into healthcare systems. This can be achieved through policy changes that emphasize the inclusion of preconception care services as a standard part of reproductive healthcare. Policymakers should also allocate resources to support the implementation of preconception care programs and initiatives, ensuring that healthcare providers have the necessary tools, guidelines, and support to deliver high-quality preconception care to individuals. Individuals themselves must take an active role in prioritizing preconception care. They should be empowered with knowledge about the significance of preconception health and educated on the steps they can take to optimize their own health and the health of their future children. This involves seeking preconception care services, engaging in healthy lifestyle choices, such as maintaining a balanced diet, exercising regularly, avoiding harmful substances, and managing existing medical conditions. By actively participating in preconception care, individuals can significantly improve their chances of having a healthy pregnancy, reducing the risk of complications, and promoting the long-term health and well-being of their children.
